# Redox Control in Platelet Activity and Therapy

**DOI:** 10.3390/antiox14111286

**Published:** 2025-10-27

**Authors:** Laura M. Dionisio, Yi Zheng, Jose A. Cancelas

**Affiliations:** 1 Division of Bone Marrow Transplantation and Cell Therapies, Department of Medical Oncology, Dana-Farber Cancer Institute, Harvard Medical School, Boston, MA 02115, USA; laura_dionisio@dfci.harvard.edu; 2 Division of Experimental Hematology and Cancer Biology, Cincinnati Children’s Hospital Medical Center, Cincinnati, OH 45229, USA; yi.zheng@cchmc.org; 3 Department of Pediatrics, University of Cincinnati College of Medicine, Cincinnati, OH 45267, USA; 4 Connell and O’Reilly Families Cell Manipulation Core Facility, Dana-Farber Cancer Institute, Harvard Medical School, Boston, MA 02115, USA; 5 Department of Medicine, Harvard Medical School, Boston, MA 02115, USA

**Keywords:** redox regulation, platelet, activity, therapy

## Abstract

Maintaining redox balance is essential for platelet physiology and overall cellular homeostasis. Upon activation, platelets generate reactive oxygen species (ROS), which act as signaling mediators in responses to collagen and are required for collagen-dependent thrombus formation. Multiple enzymatic systems contribute to platelet ROS production, with nicotinamide adenine dinucleotide (phosphate) oxidases (NOX isoforms) serving as the primary source, complemented by cyclooxygenase (COX), xanthine oxidase (XO), and the mitochondrial respiratory chain. Both oxidative and reductive stress disrupt this equilibrium and have been implicated in the pathophysiology of diverse diseases, including bleeding disorders, thrombosis, cardiovascular disorders, diabetes and cancer. In transfusion medicine, mitochondrial dysfunction and the resulting oxidative stress are key drivers of platelet lesion resulting in clearance defects and the progressive loss of hemostatic activity during storage. Targeting platelet-specific redox regulatory pathways represents a promising strategy to better define platelet contributions to human health and to develop interventions that may alter disease outcomes in which platelets play a central role.

## 1. Introduction

Platelets are small, anucleate blood cells derived from megakaryocytes whose primary function is in hemostasis, with increasingly recognized active roles in innate immunity, inflammation, wound healing and diverse disease processes. Platelets are metabolically active and highly versatile, displaying flexible use of fuels and pathways [1].

Oxidation and reduction (redox) reactions are essential for mammalian cells to generate energy and synthesize the macromolecules required for biological function [2]. In these reactions, electrons flow from reducing agents (reductants) to oxidizing agents (oxidants). Electron transfer underlies nearly all cellular metabolism processes, including the biosynthesis of amino acids, fatty acids, carbohydrates, and nucleic acids [3].

A reducing agent and its oxidizing counterpart form a redox complex. The major redox complexes in biological systems are nicotinamide adenine dinucleotide (NAD+)/reduced NAD+ (NADH), phosphorylated NAD+ (NADP+)/reduced NADP+ (NADPH), and reduced glutathione (GSH)/GSH disulfide (GSSG) [2]. These complexes regulate the redox state and link it to energy metabolism, mitochondrial function, gene expression, and signaling pathways [2,4].

Oxidative stress arises when the production of reactive oxygen species (ROS) reactive nitrogen species (RNS) exceeds the antioxidant capacity of a biological system [5,6].

ROS and RNS are byproducts of aerobic metabolism and are generated in response to both endogenous and exogenous stimuli [7]. Major species include superoxide anion (O_2_•^−^), hydrogen peroxide (H_2_O_2_), hydroxyl radical (•OH), hydroxyl ion (OH^−^), nitric oxide (•NO), nitrogen dioxide radical (•NO_2_) and peroxynitrite (ONOO^−^) [8]. Their chemical reactivity allows them to modify proteins, lipids, and nucleic acids.

Conversely, reductive stress occurs when reductants such as NADPH, NADH, and GSH, accumulate in excess to their oxidized counterparts (NADP+, NAD+, and GSSG) [9]. Oxidative stress occurs primarily from the generation of reactive oxygen species (ROS), which are produced through several enzymatic and receptor-dependent pathways, including nicotinamide adenine dinucleotide phosphate (NADPH) oxidases (NOX), cyclooxygenase-1 (COX-1), immunoreceptor tyrosine-based activation motif (ITAM)-dependent signaling, and the mitochondrial respiratory chain. When ROS production exceeds the neutralizing capacity of antioxidant systems including the SOD/GPx reductases, oxidative stress promotes platelet activation, aggregation, and thrombus formation.

Redox homeostasis is maintained by the balance between pro-oxidant and antioxidant systems. Pro-oxidants such as ROS and RNS are produced by enzymes including NAD(P)H oxidases (NOXs) and the mitochondrial electron transport chain (ETC) [2]. Additional contributors include the endoplasmic reticulum, peroxisomes, the cytochrome P450 system, and nitric oxide synthases (NOS) [3].

Conversely, reductive stress can develop when the cellular redox balance shifts excessively toward reducing equivalents such as NADH, NADPH, and glutathione (GSH). In platelets, elevated flux through the pentose phosphate pathway and increased activity of antioxidant systems (e.g., glutathione peroxidase, thioredoxin, and peroxiredoxins) may lead to an over-reduced intracellular state. This condition can impair mitochondrial electron transport by limiting electron flow, disrupt disulfide bond formation in proteins, and paradoxically sensitize platelets to oxidative bursts once reducing capacity is overwhelmed.

At physiological levels, oxidants support critical biological functions. Low levels of ROS act as signaling molecules. For example, in innate immunity, phagocytes generate ROS within phagosomes to kill engulfed pathogens [10]. ROS and RNS also act as intracellular and paracrine second messengers, regulating antioxidant responses, DNA repair, inflammation, apoptosis, cytoskeletal remodeling, and metabolic adaptation [11]. When in excess (oxidative stress), excessive accumulation of ROS/RNS can cause direct injury to different targets, including proteins, lipids, and nucleic acids, leading to cell dysfunction and death and/or clearance [12].

Proteins can undergo oxidative modifications, such as methionine and cysteine oxidation, resulting in diverse posttranslational protein modifications. Lipids and carbohydrates can be oxidized to highly reactive intermediates that react with proteins at functional sites [13]. In particular, ROS drive lipid peroxidation, attacking polyunsaturated fatty acids and generating 4-hydroxynonenal and related products that trigger apoptosis, autophagy, and ferroptosis [14]. Oxidative damage to nucleic acids has been linked to cancer [15], neurodegenerative disorders [16], chronic kidney disease [17], and cardiovascular disease [18].

As platelets are known to both ROS sources and targets, the significant role of ROS in platelet biology has been increasingly explored in recent decades, in the context of disease, therapeutics and transfusion medicine [7,19,20,21]. Reductive stress significantly impacts the development of diseases, and the most well characterized examples are diabetes and cardiovascular diseases [9].

The aim of this review is to summarize the current understanding of the mechanisms for generation and control of platelet ROS and an overview of its practical importance for health and disease.

## 2. Molecular Mechanisms of Oxidative Regulation in Platelets

**Figure 1:**
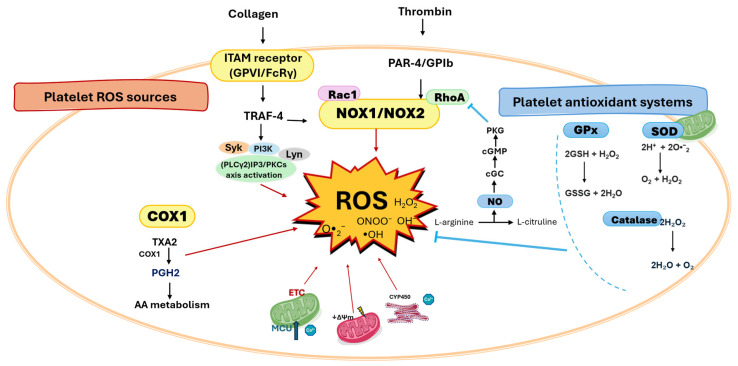
Platelet redox circuitry. The principal sources of reactive oxygen species (ROS) in platelets include NADPH oxidases 1/2 (NOX1/2), cyclooxygenase-1 (COX-1), immunoreceptor tyrosine-based activation motif (ITAM) receptors (activated by collagen), and protease-activated receptor 4 and GPIb (PAR-4/GPIb, activated by thrombin). Mitochondria generate ROS predominantly through the electron transport chain (ETC). COX-1 catalyzes the conversion of arachidonic acid (AA) to prostaglandin H2 (PGH2), a reaction that produces ROS as by-products; PGH2 is further metabolized to thromboxane A2 (TXA2), which amplifies platelet activation. The mitochondrial calcium uniporter acts as a critical regulator of ROS production in ITAM receptor-mediated platelet activation. ROS can damage mitochondria, leading to loss of mitochondrial membrane potential (ΔΨm) and further ROS generation. Key signaling hubs for ROS production in activated platelets include tumor necrosis factor receptor-associated factor 4 (TRAF4), Syk, phospholipase Cγ2 (PLCγ2)/inositol 1,4,5-trisphosphate (IP3), Ca^2+^, and Src family kinases such as Lyn. Antioxidant defenses are provided mainly by glutathione peroxidase (GPx), superoxide dismutase (SOD), and catalase. The balance of these redox processes governs platelet outcomes, including activation, acquisition of a procoagulant phenotype, and apoptosis.

**Figure 2:**
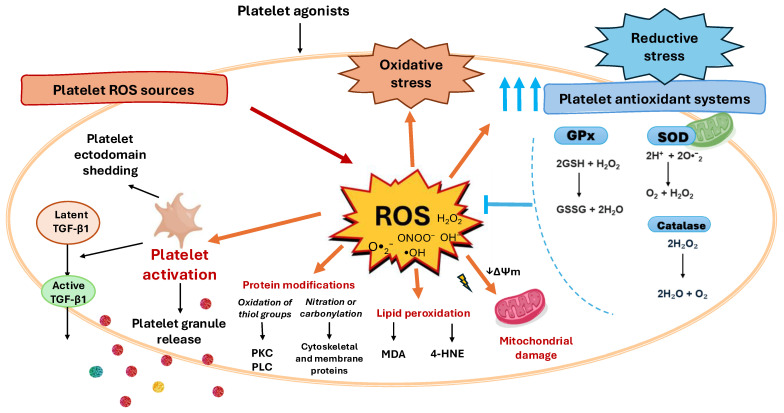
Platelet redox imbalance consequences. The balance of the redox processes within platelets governs platelet outcomes. When platelets face conditions that increase ROS production, antioxidant defenses act to restore redox balance. However, when this buffering capacity is exceeded, redox homeostasis is lost, leading to either oxidative or reductive stress. When ROS production exceeds the neutralizing capacity of antioxidant systems oxidative stress promotes platelet mitochondrial damage, platelet activation and granule release, platelet ectodomain shedding, acquisition of a procoagulant phenotype, and apoptosis. In activated platelets, ROS also mediate the release and activation of latent transforming growth factor-β1 (TGF-β1), a key profibrotic cytokine. Conversely, reductive stress can develop when the cellular redox balance shifts excessively toward reducing equivalents such as NADH, NADPH, and glutathione (GSH), which, paradoxically, can induce further ROS production, thus amplifying the oxidative damage, a phenomenon termed the antioxidant paradox. Among the targets of ROS generated within platelets are also lipids and proteins, while lipids can undergo lipid peroxidation, that generates reactive aldehydes such as malondialdehyde (MDA) and 4-hydroxynonenal (4-HNE), which further propagate oxidative stress, proteins amino acidic residues are subject to oxidative modifications, such as the oxidation of Sulphur-containing residues, affecting, for example, the activity of signaling enzymes such as protein kinase C (PKC) and phospholipase C (PLC), while nitration or carbonylation of cytoskeletal and membrane proteins can alter platelet shape change and aggregation.

The main molecular causal mechanisms of activation of ROS and antioxidant systems present in platelets are categorized, described below and summarized in Figure 1, while the consequences of platelet redox imbalance are presented in Figure 2.

### 2.1. Oxidative Stress Regulators

#### 2.1.1. NADPH Oxidases (NOX)

NADPH oxidases (NOX) are considered the main enzymatic source of ROS in platelets. These enzymes catalyze the transfer of electrons from NADPH across membranes to molecular oxygen, generating O_2_•^−^ [7].

In resting platelets, NOX1 and NOX2 are expressed in an inactive state. Upon platelet activation, they assemble with cytoplasmic regulatory subunits, including p40^phox^, p47^phox^, and p67^phox^, forming the functional NOX complex [22,23]. The recruitment of small GTPases Rac1 or Rac2 to p67^phox^ promotes a conformational change that is essential for full enzyme activation [24]. The resulting increase in ROS production amplifies platelet adhesion, aggregation, and signaling.

Evidence for the role of NOX in platelet activation also comes from their role in extracellular vesicle (EV) activity. Activated platelets release EVs enriched in NOX1 and its regulatory components; these EVs can, in turn, stimulate further platelet activation. Pharmacologic inhibition of NOX1 abrogates this effect, underscoring its functional relevance [25].

Functionally, NOX isoforms appear to regulate stimulus-specific platelet responses. In humans, NOX1 is the predominant source of ROS downstream of collagen receptor signaling, whereas NOX2 plays a critical role in thrombin-mediated platelet activation [26].

#### 2.1.2. Cyclooxygenase-1 (COX-1)

Upon platelet activation, membrane phospholipids are hydrolyzed to release arachidonic acid (AA), which is metabolized through a sequential enzymatic cascade. COX-1 catalyzes the conversion of AA to the unstable endoperoxide intermediate prostaglandin H_2_ (PGH_2_), a reaction that also generates ROS as by-products [27,28] PGH_2_ then serves as the substrate for downstream synthases, leading to the production of thromboxane A_2_ (TXA_2_) and other prostaglandins. TXA_2_ is a potent autacoid that amplifies platelet activation by promoting shape change, granule secretion, and aggregation, thereby reinforcing thrombus formation.

#### 2.1.3. ITAM Receptor

Receptors bearing immunoreceptor tyrosine-based activation motif (ITAM) sequences are critical mediators of platelet activation. Human platelets express three such receptors: (i) FcRγ, which associates with the collagen receptor glycoprotein (GP) VI (GPVI); (ii) C-type lectin-like receptor 2 (CLEC-2); and (iii) FcγRIIA, the low-affinity Fc receptor for IgG [29].

GPVI is a transmembrane receptor structurally related to immune-type receptors. Its short cytoplasmic tail lacks intrinsic signaling capacity but forms a noncovalent complex with the ITAM-containing FcRγ chain [30].

Collagen binding to GPVI triggers robust platelet activation characterized by an oxidative burst, notably increased production of H_2_O_2_, which acts as a secondary messenger by amplifying arachidonic acid metabolism and phospholipase C signaling [31].

Upon ligand engagement, GPVI-associated Src family kinases such as Lyn phosphorylate the ITAM of FcRγ, leading to recruitment and activation of spleen tyrosine kinase (Syk). A similar Syk-dependent pathway is triggered by FcγRIIA ligation [32].

Interestingly, GPVI/FcRγ signaling induces two temporally distinct phases of ROS generation in human platelets: an early burst that is Syk-independent, and a later, sustained phase that requires Syk activation [33].

Mechanistically, ITAM-driven ROS production is linked to NADPH oxidase activity through tumor necrosis factor receptor-associated factor 4 (TRAF4). TRAF4 selectively binds to the cytoplasmic tail of GPVI and interacts with the regulatory NOX subunit p47^phox^, a component of the NOX1/2 complex. This interaction provides a direct molecular link between ITAM signaling and redox-dependent platelet activation [34].

#### 2.1.4. GPIbα and Protease-Activated Receptor-4 (PAR4)

Human platelets express two protease-activated receptors, PAR1 and PAR4, which mediate thrombin-induced platelet activation [35]. In addition to PAR receptors, thrombin can also bind to GPIbα, a subunit of the von Willebrand factor receptor complex [36].

Thrombin-induced ROS generation requires coordinated signaling through both GPIbα and PAR4. Functional inhibition of either receptor abrogates thrombin-stimulated ROS production, highlighting their interdependent roles [37].

Downstream of receptor engagement, ROS production is mediated by NADPH oxidase activity, specifically NOX1, in cooperation with focal adhesion kinase (FAK) [37]. Notably, FAK also contributes to ROS generation during collagen-induced platelet activation, suggesting a shared redox-sensitive signaling axis downstream of distinct agonists [38].

#### 2.1.5. Platelet Mitochondria and Calcium

Mitochondria are the principal organelles responsible for ROS production in most cells, including platelets. Within the mitochondrial electron transport chain (ETC), leakage of electrons—particularly at complexes I and III—leads to the partial reduction of oxygen and the generation of O_2_•^−^ [39]. This mitochondrial ROS can be further converted to hydrogen peroxide (H_2_O_2_), which participates in intracellular signaling and redox regulation of platelet activation [40].

Mitochondrial ATP and ROS production are tightly coupled with oxygen availability. As the terminal electron acceptor in the mitochondrial electron transport chain (ETC), oxygen drives the continuous flow of electrons through the complexes, enabling oxidative phosphorylation. When electron transfer is incomplete or oxygen levels are limiting, electrons may prematurely react with oxygen, leading to O_2_•^−^ formation and subsequent ROS production [41].

The mitochondrial electron transport chain (ETC) is composed of five protein complexes embedded in the inner mitochondrial membrane. Complexes I–IV function as molecular pumps, transferring electrons derived from metabolic substrates while simultaneously moving protons from the matrix to the intermembrane space [42]. This proton efflux generates an electrochemical gradient—negative inside the matrix and positive in the intermembrane space—that is subsequently harnessed by Complex V (ATP synthase) to produce ATP [41]. A major source of mitochondrial ROS arises from premature electron leakage at complexes I, II, and III. These electrons reduce molecular oxygen in a one-electron reaction, forming O_2_•^−^ [42,43].

In addition to energy metabolism, mitochondria play a central role in calcium homeostasis, a key regulator of platelet activation. Calcium is taken up from the cytosol via the mitochondrial calcium uniporter (MCU) and released through the mitochondrial sodium/calcium exchanger [3]. Importantly, the MCU has been identified as a regulator of mitochondrial ROS production specifically during ITAM receptor-mediated platelet activation (e.g., collagen–GPVI/FcRγ), but not during G protein-coupled receptor signaling (e.g., ADP or thrombin) [44].

#### 2.1.6. Endoplasmic Reticulum (ER) ROS

The endoplasmic reticulum (ER) also contributes to ROS generation in platelets, primarily through the ER-resident enzymes such as NOX, ER oxidoreduction 1 (ERO1) and protein disulfide isomerase family (PDIs) [45]. During protein folding and detoxification reactions, these enzymes can transfer electrons to molecular oxygen, producing ROS as a by-product [46]. Although the overall contribution is quantitatively lower than that of mitochondrial ROS, ER-derived ROS may fulfill specialized functions in platelet stress responses and redox-sensitive signaling pathways [24].

### 2.2. Regulation of ROS Generation–Antioxidant Mechanisms in Platelets

Platelets contain several endogenous antioxidant systems that limit excessive ROS production and prevent oxidative stress-induced platelet dysfunction. The primary enzymatic system controlling ROS generation in platelets include glutathione peroxidases (GPx), superoxide dismutases (SOD) and catalase [47]. Additional regulators such as Rho-family small GTPases and zinc signaling also play important roles in maintaining redox homeostasis.

#### 2.2.1. Glutathione Peroxidases (GPx)

GPx are selenium-dependent antioxidant enzymes present in the cytoplasm and mitochondria of platelets. They catalyze the reduction of hydrogen peroxide (H_2_O_2_) and lipid hydroperoxides, using glutathione (GSH) as a co-substrate, which is oxidized to glutathione disulfide (GSSG) [48]. The recycling of GSSG back to GSH is mediated by glutathione reductase in an NADPH-dependent process, ensuring continuous antioxidant activity [49].

Human platelets express four GPx isoforms: GPx1, GPx3, GPx4, and GPx7 [50]. These enzymes localize to membranes, mitochondria, and cytosol, but their distribution changes upon platelet activation. Activated platelets show increased GPx localization to membranes, suggesting a protective role at sites of high ROS flux, such as during vascular injury [51].

GPx-3, an extracellular isoform, is particularly relevant for platelet redox regulation. In murine models, GPx-3 deficiency leads to enhanced platelet-dependent thrombosis and vascular dysfunction due to impaired ROS homeostasis and reduced nitric oxide (NO)-mediated platelet inhibition [52]. Clinically, GPx-3 deficiency is associated with a prothrombotic state in humans; Freedman et al. demonstrated that pediatric patients with cerebral thrombotic disorders and reduced plasma GPx activity exhibited impaired NO-mediated platelet inhibition, which was restored by supplementation with exogenous GPx [53].

#### 2.2.2. SOD and Catalase

SOD catalyzes the dismutation of O_2_•^−^ into H_2_O_2_, which is then further detoxified by catalase, GPx, or peroxiredoxins [54]. In mammals, three isoforms exist: SOD1 (cytosolic, also present in the mitochondrial intermembrane space), SOD2 (mitochondrial matrix), and SOD3 (extracellular). Platelets express SOD1 and SOD2 [55,56]. Of relevance is SOD2. Although platelet-specific deletion of SOD2 in mice does not impair primary hemostatic functions such as adhesion and aggregation, it markedly alters mitochondrial function, ROS production, calcium handling, thrombin generation, and predisposes to arterial thrombosis during aging [57].

Catalase, expressed at an estimated 12,000 copies per platelet, catalyzes the breakdown of H_2_O_2_ into water and oxygen without consuming reducing equivalents [50]. Functionally, catalase attenuates collagen-induced platelet activation by scavenging H_2_O_2_, thereby limiting the secondary activation of arachidonic acid metabolism and PLC signaling [31]. This highlights its protective role against excessive H_2_O_2_ accumulation and platelet hyperactivation.

#### 2.2.3. Rho-Subfamily of Small GTPases and Their Signaling

Rho GTPases (including Rac1, Cdc42, and RhoA) are small signaling proteins that cycle between inactive GDP-bound and active GTP-bound states, regulated positively by guanine nucleotide exchange factors (GEFs), and negatively by GTPase-activating proteins (GAPs), and GDP dissociation inhibitors (GDIs) [58,59]. In platelets, they regulate cytoskeletal remodeling, secretion, spreading, and thrombus formation, and are also important in late stages of megakaryopoiesis [58,60,61].

In platelets, Rac1 promotes NOX1 activation through binding to p67^phox^ in response to thrombin, GPVI, and GPIb agonists, whereas RhoA negatively regulates thrombin-induced ROS generation by suppressing p47^phox^ phosphorylation [62,63].The functional significance of this regulation is underscored by genetic and pharmacological studies. Deletion of Rac1 in mouse platelets or pharmacological inhibition of Rac1 in human platelets significantly reduced collagen-mediated ROS production [62]. Similarly, treatment of human platelets with the small-molecule RhoA inhibitor Rhosin, or selective deletion of RhoA in mouse platelets, led to reduced ROS generation in response to thrombin, highlighting a key role for RhoA in agonist-induced ROS production [63]. In turn, ROS can directly activate RhoA, independently of canonical regulatory proteins, through oxidation of two critical cysteine residues located in a unique redox-sensitive motifs [64,65]. Overall, Rho GTPases can both regulate and are regulated by ROS. They influence cellular redox balance by modulating enzymes that produce or detoxify ROS and RNS [60,66].

#### 2.2.4. Zinc

Zinc is the second most abundant transition metal in the human body after iron. It is an essential structural component of numerous enzymes, particularly intracellular metalloproteins [67], and plays critical roles in both normal cellular function and the pathophysiology of diseases such as atherosclerosis [68].

Zinc supplementation has demonstrated antioxidant effects [69,70,71], serving as a cofactor for the antioxidant enzyme superoxide dismutase (SOD) [72], and as a regulator of glutathione peroxidase (GPx) activity [73]. Additionally, zinc exerts cytoprotective effects by stabilizing cell membranes and limiting oxidative damage [74]. Platelets store zinc ions (Zn^2+^) within α-granules and the cytoplasm and release them upon activation. Extracellular zinc contributes to coagulation, platelet aggregation, and fibrin clot formation [75,76]. Conversely, zinc deficiency impairs platelet aggregation and calcium uptake—defects that can be reversed by glutathione (GSH) supplementation—highlighting zinc’s role in maintaining platelet redox balance [74]. Increases in cytosolic Zn^2+^, whether derived from extracellular sources or agonist-induced mobilization, stimulate NADPH oxidase and mitochondrial ROS production. The resulting ROS generation activates MAPK signaling pathways, including ERK1/2 and JNK, which further amplify oxidative signaling in a positive feedback loop [77]. Thus, zinc serves as both a mediator of hemostasis and a modulator of redox signaling in platelets.

#### 2.2.5. Magnesium (Mg^2+^)

Mg^2+^ is an essential nutrient for cardiovascular health, and its deficiency has been linked to the development of atherosclerosis [78]. At the intracellular level, Mg^2+^ deficiency induces mitochondrial dysfunction, leading to increased mitochondrial reactive oxygen species (ROS) production and consequent oxidative stress [79]. Conversely, Mg^2+^ supplementation has been shown to reduce mitochondrial ROS generation [80]. In addition, Mg^2+^ modulates antioxidant defense systems, including superoxide dismutase (SOD) and catalase [81]. Magnesium deficiency can further exacerbate oxidative stress by inducing mitochondrial damage and depolarization of the mitochondrial membrane potential [82]. Low Mg^2+^ levels also lead to cytosolic calcium accumulation, which disrupts oxidative phosphorylation and promotes platelet activation and ROS generation [44,83].

#### 2.2.6. Platelet Nitric Oxide (NO) Peculiarities

Although endothelial cells are the primary source of NO in the circulation, platelets also express endothelial nitric oxide synthase (eNOS) [84,85], and can both release NO in small amounts and also respond to NO [68,69]. As a result, they can produce small amounts of NO and respond to NO derived from other sources. The NO-soluble guanylyl cyclase (sGC)–cyclic GMP (cGMP)/protein kinase G (PKG) signaling pathway plays a key role in inhibiting platelet activation. NO increases intracellular levels of cGMP and cyclic AMP (cAMP), which activate protein kinase G (PKG) and protein kinase A (PKA), respectively. These kinases phosphorylate multiple substrates that mediate platelet inhibition [86].

PKG inhibits RhoA [87], a critical regulator of reactive oxygen species (ROS) generation, as described in the previous section. Thus, NO acts as a regulator of both platelet activation and ROS production through this pathway. Conversely, under conditions of oxidative stress, excess superoxide rapidly inactivates NO and promotes eNOS uncoupling—that is, the diversion of oxygen reduction from NO synthesis toward superoxide generation—thereby exacerbating oxidative imbalance [72].

### 2.3. ROS-Mediated Modifications of Key Proteins and Lipids in Platelets

ROS can induce covalent modifications of critical platelet proteins and membrane lipids, thereby altering signaling pathways, receptor function, and platelet reactivity. ROS mediate oxidative modifications of essential platelet proteins and lipids, profoundly influencing platelet function. Oxidation of thiol groups in cysteine residues affects the activity of signaling enzymes such as protein kinase C (PKC) and phospholipase C (PLC), while nitration or carbonylation of cytoskeletal and membrane proteins can alter platelet shape change and aggregation [88]. Similarly, lipid peroxidation generates reactive aldehydes such as malondialdehyde (MDA) and 4-hydroxynonenal (4-HNE), which further propagate oxidative stress and modulate receptor function and membrane fluidity [89]. These ROS-dependent molecular modifications collectively contribute to platelet hyperreactivity under pathological conditions such as diabetes, atherosclerosis, and thrombosis.

### 2.4. Oxidative Stress During Platelet Storage

During storage, platelets undergo progressive morphological, biochemical, and functional alterations, collectively termed platelet storage lesions (PSL), which limit their shelf life [90]. Human platelet concentrates gradually lose hemostatic function while acquiring enhanced immunomodulatory properties over time. When transfused into mice, aged platelet concentrates have been shown to amplify inflammatory responses and tissue injury, including the development of acute lung injury [91].

Oxidative stress and mitochondrial damage are key contributors to PSL, and the presence of mitochondrial DNA released from damaged mitochondria in platelet concentrates increases over storage time in correlation with ROS levels [92].

Several studies have reported that intraplatelet ROS formation rises significantly after day 3 of storage [93,94]. Importantly, ROS-induced signaling in stored platelets can act as a mediator of platelet activation and inflammation. Elevated ROS levels are associated with platelet granule release and a pro-inflammatory phenotype, as platelets with higher ROS display increased expression of irreversible activation markers such as P-selectin and CD40 L, whereas P-selectin-negative platelets contribute minimally to ROS production [93].

ROS generation during storage is also linked to the loss of adhesive receptor function. Increased ROS promotes ectodomain shedding of GPVI and GPIbα and the formation of microparticles [95], both established hallmarks of PSL [96,97]. This loss of adhesive receptors impairs platelet adhesion, since platelets with elevated ROS show reduced capacity to bind collagen matrices [95].

Autophagy, an essential catabolic process for maintaining intracellular homeostasis by degrading protein aggregates and damaged organelles, also declines during platelet storage [98,99]. A slow decay of autophagic flux is observed, and pharmacological manipulation of autophagy alters redox balance: treatment with the autophagy inducer rapamycin reduces platelet ROS generation and receptor shedding [98].

Given the short shelf life of platelet concentrates, cold-stored platelets (CSP) are being explored as an alternative to extend storage time and lower post-transfusion infection risks. However, cold storage itself induces PSL, particularly during prolonged storage [19,100].

Cold exposure promotes platelet activation, cytoskeletal reorganization, cell surface protein clustering, impaired fibrinogen spreading, mitochondrial metabolic uncoupling with enhanced ROS generation, and accelerated clearance by both macrophage-dependent phagocytosis and macrophage-independent pathways [19,100,101]. The contribution of ROS to cold-induced PSL is underscored by findings that supplementation with the antioxidant N-acetylcysteine (NAC) preserves platelet function and prevents premature clearance in vitro and in pre-clinical animal models [19]. More recently, RhoA GTPase has been identified as a key molecular switch driving cold storage lesions. Cold-induced activation of RhoA reproduces the main features of cold storage injury, while selective RhoA inhibition in cold-stored platelet concentrates reduces ROS generation and ameliorates PSL [102].

**Figure 3:**
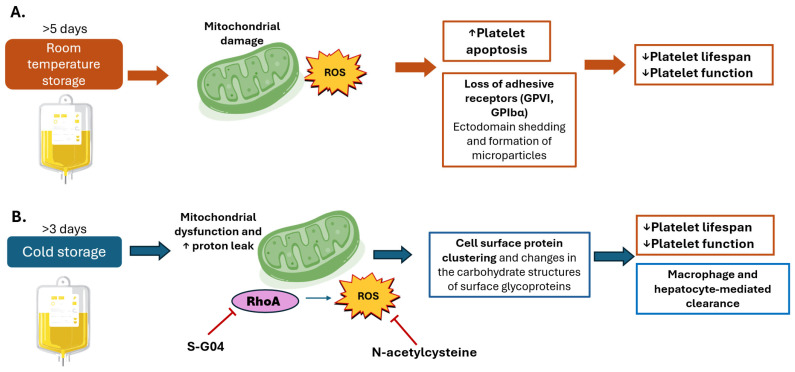
Oxidative damage during platelet storage. Mechanisms and consequences of platelet storage during (**A**) room temperature and (**B**) cold storage. (**A**) Room temperature storage (20–24 °C), which remains the standard practice in trans-fusion medicine because it preserves platelet viability and recovery for several days after transfusion. Prolonged storage induces a series of metabolic, structural, and functional changes known collectively as the platelet storage lesion. One of the earliest events is mitochondrial dysfunction: mitochondria progressively lose membrane potential, leading to impaired oxidative phosphorylation and a shift toward glycolytic metabolism. This metabolic reprogramming is accompanied by excessive production of reactive oxygen species (ROS), which overwhelm intrinsic antioxidant defenses. ROS act as both damaging molecules and signaling mediators, triggering apoptotic pathways through caspase activation, phosphatidylserine exposure, and mitochondrial permeability transition. Simultaneously, oxidative stress promotes ectodomain shedding of key adhesion receptors such as glycoprotein VI (GPVI) and glycoprotein Ibα (GPIbα). The loss of these receptors directly reduces platelet ability to adhere to subendothelial collagen and von Willebrand factor, impairing clot formation. Moreover, shedding and apoptosis are coupled with the release of platelet-derived microparticles, which contribute to proinflammatory and procoagulant signaling in the circulation. Structural changes, including transition from a discoid to spherical morphology and cytoskeletal rearrangements, further compromise platelet functionality. Collectively, these events culminate in shortened platelet lifespan, diminished hemostatic efficacy, and reduced transfusion benefit. (**B**) Cold storage (1–6 °C). Cold storage, once abandoned due to rapid platelet clearance, has regained interest for its potential advantages in trauma and acute bleeding settings, where immediate hemostatic function is prioritized over circulation time. However, cooling imposes unique stresses on platelet physiology. At the membrane level, cold temperatures disrupt lipid bilayer organization, induce clustering of glycoprotein receptors, and trigger cytoskeletal remodeling, which together alter platelet morphology and increase activation propensity. Mitochondria are also directly affected. Cold exposure promotes uncoupling of oxidative phosphorylation, characterized by proton leak across the inner mitochondrial membrane. This uncoupling increases ROS generation, compromising bioenergetic efficiency and amplifying oxidative stress. The resulting ROS act as a signal for accelerated recognition and clearance of transfused platelets by hepatic macrophages (Kupffer cells) and hepatocytes. Consequently, cold-stored platelets exhibit markedly reduced circulation time, limiting their utility in prophylactic transfusions. Emerging interventions aim to mitigate these adverse effects. Antioxidants such as NAC reduce cold-induced oxidative stress, while pharmacologic inhibition of RhoA GTPase with S-G04 has been shown to preserve platelet function and delay clearance. These strategies highlight the possibility of extending platelet lifespan under cold storage without compromising their enhanced immediate hemostatic activity. Upwards arrows denote increased effect. Downwards arrows denote decreased effect.

The mechanisms and major consequences of PSL during room temperature and cold storage are summarized in Figure 3.

## 3. Reductive Balance of the Redox System in Platelets

Under physiological conditions, cellular redox systems maintain oxidants and reductants within a narrow range. When cells face conditions that increase ROS production, antioxidant defenses act to restore balance. However, once this buffering capacity is exceeded, redox homeostasis is lost, leading to either oxidative or reductive stress. Reductive stress is defined by abnormally low ROS levels that impair their essential signaling functions [2].

Whereas oxidative stress has been widely studied, reductive stress has is a relatively underexplored aspect of cellular redox biology [103]. In platelets, ROS generated upon stimulation by agonists act as second messengers that amplify activation responses [7].

Consequently, insufficient ROS due to reductive stress can compromise platelet function. A growing body of evidence suggests that a lack of ROS can be as detrimental as their excess, leading to harmful disturbances in redox signaling [103,104,105,106,107].

The NADH/NAD^+^ couple serves as a central hub in cellular redox metabolism, including in platelets [22,23]. Reductive stress is characterized by elevated NADH/NAD^+^ and NADPH/NADP^+^ ratios and persistent activation of antioxidant defenses [106]. NADH reductive stress arises when NADH production outpaces consumption, a scenario that may result from mitochondrial dysfunction, hypoxia, overnutrition, or defective metabolic enzymes [105,108,109,110]. This imbalance drives metabolic reprogramming across glucose, lipid, amino acid, and nucleotide pathways [104].

Paradoxically, reductive stress can also promote oxidative damage, a phenomenon termed the *antioxidant paradox* [103,111]. When mitochondrial complexes are hyper-reduced, excess electrons leak to oxygen, generating O_2_•^−^ [103]. In platelets, this paradox was illustrated by studies with resveratrol, a natural antioxidant tested to prevent storage lesions. Resveratrol suppressed collagen-induced responses and inhibited aggregation in a dose-dependent manner. At low concentrations (10 μM), resveratrol effectively controlled ROS levels, but higher concentrations unexpectedly increased ROS production [112]. This effect likely reflects its capacity to upregulate endogenous antioxidant enzymes such as SOD, GPx, and catalase, thereby inducing reductive stress [113].

A similar biphasic effect was observed with NAC. While low NAC concentrations reduced ROS generation and platelet clearance during cold storage [19], high concentrations facilitated mitochondrial ROS formation and induced platelet activation [114], which may explain the discrepancy between pre-clinical animal models and results in clinical trials [115]. Together, these findings indicate that excessive antioxidant activity can paradoxically trigger ROS production through reductive stress.

Thus, the challenge in using antioxidants to preserve platelet function lies in identifying a therapeutic window that suppresses harmful oxidative stress without tipping the balance toward reductive stress, which may exacerbate ROS-mediated damage.

## 4. Oxidative Stress in Platelets in the Context of Diseases

Multiple mechanisms have been proposed to explain the interplay between platelets and ROS in the pathophysiology of numerous diseases. Across diverse pathological conditions, impaired ROS homeostasis consistently associates with platelet hyperactivity, as described below.

### 4.1. Immune Thrombocytopenic Purpura

A role for oxidative stress in the pathophysiology of immune thrombocytopenic purpura (ITP) has been investigated by several studies. In pediatric patients with acute and chronic ITP, oxidative stress markers such as serum malondialdehyde (MDA) and total oxidant status (TOS) are significantly increased compared with healthy controls, while serum total antioxidant capacity TAC level is reduced, reflecting a state of redox imbalance in the disease [116]. Patients in remission show a significant increase in TAC, catalase, and GSH levels when compared with baseline (pre-treatment), although GPx activity remains elevated during both active disease and remission [117,118].

Trace elements also appear to contribute to the redox imbalance. Levels of serum copper and zinc, which are essential for antioxidant enzyme function, are reduced system are also decreased in patients with ITP compared with healthy controls. Moreover, patients with relapsed and treatment-refractory ITP display lower serum copper levels than treatment-responsive patients [119].

More recently, Wang et al. reported that oxidative stress in platelets from ITP patients activation and assembly of the NOD-like receptor pyrin 3 (NLRP3) inflammasome, which may drive disease pathogenesis of ITP [120]. NLRP3, a multiprotein complex activated by a variety of “cellular danger” signals including oxidative stress [121], initiates hydrolysis of pro-caspase-1 to active caspase-1, leading to IL-1β maturation and pyroptosis, a pro-inflammatory form of cell death [122].

### 4.2. Thrombosis

Thrombosis is a leading cause of mortality worldwide and underlies most heart attacks, strokes, and venous thromboembolism (VTE), which includes pulmonary embolism (PE) and deep vein thrombosis (DVT) [123]. Thrombosis is a multifactorial process involving endothelial cells, platelets, and red blood cells, whose functions can be altered by oxidative stress and inflammation, initiating events that promote thrombus formation [124]. Both excessive intraplatelet ROS production and impaired antioxidant defenses contribute to thrombotic risk, as discussed below.

The mitochondrial antioxidant enzyme superoxide dismutase 2 (SOD2) is a critical regulator of platelet ROS. Studies in aged mice deficient in platelet SOD2 demonstrated that loss of this enzyme exacerbates age-related increases in intraplatelet ROS, calcium elevation, mitochondrial hyperpolarization, annexin V binding, and platelet-dependent thrombin generation. Consequently, SOD2 deficiency heightened susceptibility to arterial thrombosis [57].

Platelet mitochondria also play a crucial role in thrombus formation through the generation of procoagulant platelets. When exposed to oxidative stress, ROS-damaged mitochondria undergo the opening of the mitochondrial permeability transition pore (mPTP), a nonselective multiprotein channel spanning the inner mitochondrial membrane, which leads to a rapid loss of mitochondrial membrane potential [125]. This loss of potential serves as a key signal regulating the externalization of phosphatidylserine on the platelet surface [126], a defining feature of the procoagulant platelet phenotype [127].

In parallel, age-related increases in ROS also activate mechanistic target of rapamycin complex 1 (mTORC1) in megakaryocytes and platelets [128]. The mTOR pathway is central to cellular and physiological homeostasis, and its dysregulation is linked to metabolic disease, cancer, and aging [129]. In aged mice, ROS-mediated mTORC1 activation enlarged megakaryocyte and platelet size, promoted platelet activation, and increased the incidence of venous thrombosis [128].

As detailed in Section 2, platelets express multiple NOX isoforms. NOX1 and NOX2 participate in distinct activation pathways, but both generate ROS that enhance platelet activation through a common mechanism involving Syk/phospholipase Cγ2/calcium signaling. Importantly, NOX2, but not NOX1, is required for platelet thrombus formation at sites of vascular injury [130].

Clinical studies further support the role of redox imbalance in thrombosis. Platelets from PE patients exhibited elevated mitochondrial ROS, reduced mitochondrial anti-apoptotic protein Bcl-2, and increased cytosolic cytochrome c. These changes coincided with phosphatidylserine exposure and P-selectin expression, consistent with platelet hyperactivation and apoptosis, contributing to the hypercoagulable state in PE [131].

Pharmacological inhibition of NOX enzymes has shown promise in mitigating platelet-driven thrombosis. Both a pan-NOX inhibitor [21] and a dual NOX1/4 inhibitor [132] reduced ROS production, platelet activation, and thrombus formation. In addition, antioxidants from various sources have been reported to decrease platelet activation and protect against thrombosis [133,134].

Together, these findings highlight redox imbalance as a key mediator of platelet activation and thrombosis, and they underscore the therapeutic potential of targeting ROS-generating enzymes or bolstering antioxidant systems to reduce thrombotic risk.

### 4.3. Cancer

The interactions between platelets and cancer cells are described to be bidirectional [135]. Cancer progression is influenced by both platelet activity and ROS, which are integral components of the tumor microenvironment. Upon activation, platelets release cytokines and growth factors that stimulate ROS production and influence tumor biology. Conversely, cancer cells, driven by their accelerated metabolism to sustain rapid proliferation, generate high levels of ROS, which in turn alter platelet phenotype and function [15].

Beyond paracrine signaling, platelets may directly promote metastasis by directly transferring mitochondria to cancer cells. This process mediated by the PINK1/Parkin–Mfn2 pathway enables platelet-derived mitochondria to reprogram osteosarcoma cells toward a metastatic state. The transferred mitochondria lower cancer cell ROS levels by modulating the cancer cell GSH/GSSG ratio, thereby facilitating metabolic shifts favorable for survival and dissemination. These findings suggest that targeting platelet mitochondrial transfer could represent a novel therapeutic strategy for preventing metastasis [136].

Although oxidative stress is a well characterized feature of cancer [137] and has been extensively investigated as an anticancer target [138,139,140], the contribution of platelet of oxidative stress to tumor biology and its therapeutic potential remain poorly explored. For example, in patients with invasive breast cancer, platelet membrane glycoproteins exhibited increases nitrative modifications, such as enhanced 3-nitrotyrosine content, compared with healthy controls, reflecting a state of redox imbalance. Notably, supplementation with a polyphenol-rich extract of *Aronia melanocarpa* attenuated this redox stress [141].

In another study, pre-surgical urinary levels of the oxidative stress markers 8-iso-prostaglandin (PG)F_2α_ and 11-dehydro-thromboxane (TX)B_2_ was observed, correlating with tumor estrogen receptor (ER) expression. These findings suggest that estrogen-driven oxidative mechanisms may contribute to persistent platelet activation in breast cancer [142].

In hematologic malignancies, platelet-derived reactive oxygen species (ROS) also contribute to disease progression. In essential thrombocythemia (ET), patient-derived platelets exhibit elevated ROS levels compared with those from healthy controls, promoting platelet activation, apoptosis, and fibrosis through activation of the JAK–STAT signaling pathway. This oxidative signaling enhances the procoagulant function of platelets. Notably, treatment with the antioxidant N-acetylcysteine (NAC) restored platelet function and reduced platelet production in ET, underscoring the pathogenic role of platelet ROS in myeloproliferative disease [143].

### 4.4. Fibrosis

ROS mediate the release and activation of latent transforming growth factor-β1 (TGF-β1), a key profibrotic cytokine. Platelets are the predominant source of circulating TGF-β1, and oxidative pathways are critical for converting latent to active TGF-β1, thereby driving pathologic fibrosis in various tissues. Thus, platelet ROS generation not only influences hemostasis and thrombosis but also contributes to fibrotic remodeling through regulation of TGF-β1 bioavailability [144].

### 4.5. Diabetes and Metabolic Diseases

Patients with diabetes mellitus (DM) display impaired redox balance, with oxidative stress serving both as a key pathogenic mechanism and as a critical link to the vascular and metabolic complications of the disease [145,146]. DM is also associated with an increased risk of thromboembolism [147,148], and the presence of a hypercoagulable state is well established [149,150]. In platelets from patients with DM, oxidative stress is characterized by increased superoxide anion production and reduced NOS activity. Together, these changes enhance agonist-induced intracellular Ca^2+^ signaling and promote platelet hyperreactivity [151,152]. Clinically, this hyperreactivity translates into an increased risk of major adverse cardiac events, including cardiac death, myocardial infarction, and stent thrombosis [153]. The mechanisms underlying increased platelet reactivity in DM are multifactorial and include contributions from hyperglycemia and ER stress [154,155].

Hyperglycemia induces the glycation of LDL particles, promoting structural and compositional modifications that increase their susceptibility to oxidation. The interaction of glycated LDL with platelets significantly augments NO production, intracellular Ca^+2^ levels and platelet reactivity to ADP, key elements driving vascular complications in DM [156]. Consistently, proteomic analysis of platelet lysates from DM patients revealed enrichment of proteins involved in response to oxidative stress responses and detoxification, such as GPX7 and selenoprotein P (SEPP1), supporting the concept that oxidative stress is a defining feature of the diabetic platelet phenotype [155].

At the megakaryocyte level, type 2 DM is associated with oxidative stress-induced damage accompanied by upregulation of mitochondrial enzymes. This oxidative stress signature is transmitted to circulating platelets where it is linked to diminished mitochondrial contributions to energy metabolism [157]. In parallel, autophagy—and in particular mitophagy—is reported to be highly upregulated in DM platelets, acting as an intrinsic protective mechanism that limits oxidative stress-induced apoptosis and thrombosis [158].

Therapeutic interventions targeting oxidative stress have shown promise. Treatment of DM patients with α-lipoic acid (ALA), a potent antioxidant with antiplatelet activity and lipid-modulating capacity prevents microvascular complications, thereby reinforcing the central role of oxidative stress in platelet hyperreactivity and its consequences in DM [159].

### 4.6. Cardiovascular Disease/Hyperlipidemia/Atherosclerosis

Hyperlipidemia particularly elevated low-density lipoprotein (LDL), is a well-characterized risk factor for atherogenesis and cardiovascular disease [160]. Hypercholesterolemia promotes platelet hyperreactivity primarily through interactions between oxidized LDL (oxLDL) and the platelet membrane. Activated platelets, in turn, contribute to oxLDL generation, establishing a feed-forward loop that sustains platelet activation and thrombus formation via ROS-dependent mechanisms [161].

LDL circulates in both native (nLDL) and oxidized (oxLDL) forms. OxLDL acts as an endogenous damage-associated molecular pattern (DAMP) recognized by platelet receptors CD36 and lectin-like oxLDL receptor-1 (LOX-1), triggering ROS production and platelet activation [47]. Activated platelets further amplify this process by converting nLDL into oxLDL, reinforcing platelet hyperactivation [162]. In this context, NOX2-derived ROS play a dual role: they drive LDL oxidation while simultaneously acting as intracellular signals that potentiate oxLDL-mediated platelet activation [163].

Alterations in the platelet lipidome may also contribute to thrombotic predisposition in coronary artery disease. One proposed mechanism involves CXCL12 and its receptors CXCR4 and CXCR7, which regulate lipid uptake in platelets [164].

Evidence for platelet-dependent LDL modification in atherosclerosis comes from studies in patients with hereditary gp91phox deficiency, a critical subunit of the NADPH oxidase complex. Platelets from these patients exhibited reduced ROS production and weak LDL oxidation, whereas platelets from hypercholesterolemic patients generated increased ROS and produced more oxLDL than those from healthy controls [165].

Mitochondrial oxidative stress also contributes to cardiovascular pathology. ROS-mediated mitochondrial DNA damage and dysfunction in platelets have been linked to ischemic events, including heart failure (HF) [166]. In HF patients, platelet NOX2 expression is upregulated, likely driven by chronic inflammation and TNFα overexpression, further enhancing ROS generation [167]. Interestingly, platelets can also exert cardioprotective effects. During acute myocardial infarction, platelets mitigate ischemia/reperfusion injury through several mechanisms, including the release of platelet-activating factor (PAF), which protects cardiomyocytes in a ROS- and mitochondria-dependent manner [168].

### 4.7. Infections

NADPH oxidase-derived ROS play a detrimental role in sepsis-induced platelet dysfunction. In septic mice, intravital microscopy revealed that platelet ROS promote abnormal adhesion and impaired blood flow in capillaries [169]. Beyond vascular effects, platelets also act as mediators of sepsis-associated acute kidney injury (SA-AKI), a frequent and severe complication in critically ill patients that is strongly linked to chronic kidney disease, cardiovascular events, and mortality [170]. In a murine sepsis model, platelets contributed to SA-AKI by inducing oxidative stress and apoptosis in renal tubular epithelial cells. Inhibition of platelet activation with ticagrelor reduced oxidative stress and renal cell apoptosis, highlighting a causal role for platelet-mediated redox imbalance in septic kidney injury [171].

Platelet-derived EVs further amplify inflammatory and oxidative damage during sepsis. EVs facilitate intercellular communication between platelets and immune or parenchymal cells [172]. When purified from lipopolysaccharide (LPS)-activated platelets, EVs exacerbated septic AKI by inducing inflammation, apoptosis, and ROS-dependent stress responses in recipient cells [173].

Impaired platelet redox homeostasis is also evident in viral infections such as COVID-19. Platelets from SARS-CoV-2-infected patients display an altered NO/ROS balance, characterized by reduced •NO production and increased ROS generation. Notably, patients who developed thrombotic complications during hospitalization exhibited significantly lower platelet NO production, suggesting that disruption of platelet redox equilibrium contributes to the prothrombotic phenotype in COVID-19 [174].

In parasitic infection-associated thrombocytopenia, platelet oxidative stress has likewise been implicated. In patients with *Plasmodium vivax* malaria, platelet antioxidant enzyme activities (SOD and GPx) were reduced, while platelet lipid peroxidation levels were elevated compared with healthy controls. Notably, platelet count negatively correlated with lipid peroxidation, linking oxidative stress to the severity of thrombocytopenia [175].

## 5. Strategies to Assess Redox Status of Platelets

Oxidative vs. reductive stress represent a “double-edged sword” in platelet biology and transfusion medicine. Strategies to modify the redox capacity of platelets have resulted in some promising results in both clinical and pre-clinical settings.

Given the substantial role of platelet ROS in disease pathophysiology and their impact on the quality of platelet concentrates for transfusion, significant efforts have been devoted to developing reliable methods for measuring platelet redox status. These include both adaptations of techniques used in other cell types and platelet-specific approaches.

Flow cytometry is the most widely used technique for detecting intracellular ROS, employing a variety of fluorescent probes, as reviewed by Masselli et al. [7]. A flow cytometric assay using dihydroethidium (DHE) as a redox-sensitive probe enables the detection of superoxide anion in human platelets. Using this method, thrombin, collagen-related peptide (CRP), and arachidonic acid, but not ADP, were shown to stimulate superoxide generation in a concentration-dependent manner. Additional probes extend the scope of detection: 2′,7′-dichlorodihydrofluorescein (H_2_DCFDA) for total intracellular ROS and MitoSOX™ Red for mitochondria-specific ROS [94,152,176].

Beyond flow cytometry, enzyme activity assays provide insights into ROS generation and antioxidant defense. NOX and SOD activities, and total antioxidant capacity (TAC) in plasma can be measured with commercially available ELISA kits [177]. TAC assays are widely applied to estimate extracellular non-enzymatic antioxidant levels. They rely on thermal radical generators to produce a constant flux of radicals, against which antioxidants compete, delaying probe oxidation [178]. TAC measurements have been proposed as an estimate of redox status in stored platelet units for transfusion [179].

Biochemical assays complement these approaches. Superoxide anion production can be quantified via the cytochrome C reduction method, where formation of cytochrome C levels measured spectrophotometrically at 550 nm, is inhibited by SOD [180]. Lipid peroxidation can be assessed by measuring malondialdehyde (MDA) levels, a widely recognized oxidative stress biomarker, using the thiobarbituric acid-reactive substances (TBARS) method in platelets [179,181].

Emerging techniques offer more integrated assessments. Vara et al. [26] developed a novel approach combining electron paramagnetic resonance (EPR) spectroscopy with turbidimetry, enabling simultaneous monitoring of platelet aggregation and oxygen radical generation. Similarly, electron spin resonance (ESR) spectroscopy with spin-trapping techniques has proven to be a highly sensitive method for detecting and characterizing ROS in biological systems, including dynamic changes during platelet activation [134].

An overview of the different tools that can be used for the assessment of the redox state in platelets is presented in Table 1.

## 6. Molecular Targets to Prevent Oxidative and Reductive Stress in Platelets

### 6.1. Prevention of PSL

Given the contribution of oxidative stress to the development of PSL, the use of antioxidant agents to prevent platelet damage during storage has attracted increasing attention. Both cellular and mitochondrial ROS progressively accumulate in platelet concentrates over storage time, and several ROS scavengers, including NAC, Mito-TEMPO, and resveratrol, have been reported to attenuate ROS generation and its deleterious effects [97,98,182].

Resveratrol, a natural plant-derived polyphenol with potent antioxidant properties, has shown promise in preventing PSL. It functions as an intracellular ROS scavenger and inhibits platelet apoptosis and activation during storage [190].

The Carica papaya leaf extract has been used clinically used to treat dengue-associated thrombocytopenia, has also been explored for mitigating oxidative stress-mediated PSL. This extract exerts membrane-stabilizing effects, attributed to flavonoids and other phenolic compounds, that protect against stress-induced plasma membrane damage. When supplemented in platelet concentrates, this extract increased TAC, upregulated the antioxidant enzymes SOD and catalase, and preserved platelet function [191].

Another promising compound is L-carnitine (β-hydroxy-γ-N-trimethylaminobutyric acid), an endogenous free radical scavenger. Supplementation with L-carnitine reduces platelet oxidative stress, decreases mitochondrial ROS production, and limits platelet apoptosis during storage, thereby attenuating PSL [179].

Beyond ROS scavengers, targeting ROS generation directly has also been proposed. Inhibition of NADPH oxidase (NOX) with the specific inhibitor VAS2870 reduced platelet apoptosis and enhanced platelet viability in stored concentrates [182].

### 6.2. Therapeutic Targeting of Platelet ROS in Disease

NAC, one of the most widely studied pharmacological antioxidants, has been investigated extensively for the prevention of oxidative stress-mediated diseases such as diabetes [192], cancer [193] and thrombosis [194]. In animal models of diabetes [146], NAC restored platelet antioxidant reserves, by increasing GSH content, as well as GPx-1 and SOD1 activity [192]. Similarly, in studies involving both mouse models and patients with essential thrombocythemia (ET), NAC treatment reversed elevated platelet ROS levels and platelet activation, two factors strongly associated with thrombotic risk in ET [195].

In addition to NAC, numerous natural compounds and plant-derived extracts have been studied for their antioxidant potential, offering therapeutic promise across multiple disease contexts [196].

Resveratrol, a well-characterized natural antioxidant, inhibits platelet aggregation in vitro [197]. Clinically, it has been shown to reduce thrombosis in colon cancer patients by inhibiting MAPK phosphorylation and activating the cyclic GMP/vasodilator-stimulated phosphoprotein pathway [198]. In preclinical studies, resveratrol also suppressed tumor-induced platelet activation in mouse models of non-small cell lung cancer [110].

Luteolin, a flavonoid with antioxidant, antitumor, anti-inflammatory, and cardioprotective activities, inhibits GPVI-mediated O_2_•^−^ production in platelets, enhances SOD and GPx activity, and suppresses NADPH oxidase activity [199].

Taxifolin, another flavonoid with antioxidant effects, decreases platelet adhesion, granule secretion, and aggregation in vitro in response to multiple agonists. In addition, taxifolin inhibits integrin αIIbβ3 outside-in signaling and reduces thrombus formation in mice [186].

Cinnamtannin B-1, an A-type proanthocyanidin, inhibits platelet endogenous ROS generation, Ca^2+^ mobilization, and subsequent aggregation in type 2 DM patients, suggesting potential application for preventing thrombotic complications in diabetes [200].

Matrine, a naturally occurring alkaloid with antioxidant and anti-inflammatory properties, inhibits platelet function and reduces both arterial and venous thrombosis, possibly through suppression of ROS generation, making it a potential therapeutic for thrombotic and cardiovascular diseases [201].

Finally, treatment with non-enzymatic antioxidants—including dimethylthiourea (DMTU), mannitol, and Tiron—significantly attenuated oxidative damage induced by collagen stimulation and improved survival in an experimental thrombosis model [134].

*Aronia melanocarpa* (AM) fruits (Rosaceae), rich in phenolic substances shown to have anti-inflammatory, antitumor, antioxidative and antiplatelet activities [202,203,204]. In patients with metabolic disease, supplementation with AM extract induced significant inhibition of platelet aggregation and decrease in the overall potential for coagulation, suggesting that the antioxidant effects could provide protective effects against thrombotic consequences of metabolic disease [205].

These findings highlight promising avenues for antioxidant-based therapeutic strategies in the prevention of platelet-associated disorders.

The main antioxidants described in the literature for the prevention of platelet storage lesion in addition to reported effects on platelet ROS production and thrombosis prevention in animal models and their translation potential are summarized in Table 2.

Finally, while modulation of mitochondrial proteins in platelets presents an appealing therapeutic strategy, it is essential to consider the potential systemic consequences of such interventions. Mitochondrial pathways are highly conserved and fundamental to the function of virtually all cell types, including hematopoietic progenitors, endothelial cells, and immune effector populations. Therefore, targeting mitochondrial metabolism, redox balance, or protein import machinery in platelets may inadvertently disrupt cellular energy production, calcium homeostasis, or apoptotic regulation in non-platelet compartments. These off-target effects could manifest as hematopoietic dysfunction, vascular toxicity, impaired immune responses, or altered tissue repair and regeneration. Careful evaluation of dose, specificity, and context-dependent mitochondrial vulnerabilities will thus be critical to realizing the therapeutic potential of platelet-targeted interventions while minimizing systemic adverse effects.

## 7. Effects of Anti-Platelet Therapies on Platelet Redox Balance

Although the direct effects of antiplatelet therapies on platelet redox balance have not been systematically evaluated, several antiplatelet and anticoagulant agents have demonstrated global antioxidant effects that may help prevent oxidative damage-mediated platelet dysfunction. The P2Y_12_ receptor inhibitor clopidogrel has shown significant antioxidative activity in animal models of acute myocardial infarction (AMI), resulting in decreased malondialdehyde (MDA) production and preservation of catalase activity [208]. Similarly, the novel oral anticoagulants dabigatran and rivaroxaban have been reported to reduce ROS levels and prevent DNA oxidative damage in models of vascular endothelial injury induced by 25-hydroxycholesterol (25-OHC). In AMI rabbits, dabigatran treatment significantly increased catalase and superoxide dismutase (SOD) activities while reducing inducible nitric oxide synthase (iNOS) expression [209]. Rivaroxaban, alone or in combination with aspirin, demonstrated a dose-dependent ability to suppress GPVI-mediated ROS production in platelets by attenuating Nox2 activation [210].

## 8. Conclusions and Future Directions

Platelet redox biology is central to hemostasis, thrombosis, inflammation, infection response, cancer progression, metabolic disease, and transfusion medicine. Both oxidative and reductive imbalances perturb platelet signaling and function and both can paradoxically promote ROS-dependent injury. Understanding stimulus- and compartment-specific ROS sources (NOX isoforms, mitochondria, ER) and the networks that constrain them (GPx, SOD, catalase, Rho-GTPases, zinc signaling, autophagy) offers mechanistic insight and multiple therapeutic entry points.

Despite significant advances in understanding platelet biology, several critical gaps remain in the regulation of redox signaling. They include the following: (a) Our understanding of the mechanistic specificity of ROS sources: while NADPH oxidases, mitochondria, and other enzymatic systems are recognized contributors to platelet ROS, their relative contributions under physiological versus pathological conditions remain poorly defined. (b) Regarding specific spatiotemporal dynamics of ROS signaling, it is unclear how localized versus global ROS production within platelets influences receptor signaling, granule release, and cytoskeletal reorganization during activation and clot formation. (c) Integration with metabolic pathways: the crosstalk between redox balance and platelet metabolic reprogramming (glycolysis, OXPHOS, fatty acid metabolism) is not fully delineated, limiting our understanding of how metabolic shifts shape platelet function. (d) Role of activation of reductive stress and antioxidant mechanism: platelet-intrinsic antioxidant systems (e.g., glutathione, peroxiredoxins, superoxide dismutases) are incompletely characterized, and the mechanisms by which they restore redox homeostasis after activation are not well defined. (e) Role of redox regulation in non-hemostatic functions: how redox signaling regulates platelet-mediated immune responses, inflammation, and fibrosis, particularly through cytokine release and TGF-β1 activation, remains insufficiently explored.

Key translational priorities include defining safe and effective antioxidant windows (avoiding reductive stress), refining NOX-targeted strategies, preventing PSL in stored platelets via targeted additives (antioxidants, NOX inhibitors, autophagy modulators), and exploring platelet mitochondrial transfer as a target in metastasis. The current lack of well-defined biomarkers to reliably measure platelet redox status in patients and limited strategies to selectively modulate platelet ROS without broadly affecting redox biology in other cell types limit currently proposed approaches. Standardized, platelet-specific redox assays and integrated spectroscopic approaches will accelerate progress by enabling robust, comparable measures across labs and clinical studies.

Addressing these gaps will be essential for developing targeted therapies that modulate platelet redox balance to prevent thrombotic, inflammatory, and fibrotic complications while preserving normal hemostatic function.

## Figures and Tables

**Table 1:** Summary of ROS/RNS probes and assays.

Method/References	Sample Preparation	Readout	Strengths	Limitations
Dihydroethidium (DHE) [152,182]	Platelet rich plasma (PRP) incubated for 1 h at 37 °C with the fluorescent probe	Flow cytometry	Small sample volume. Different samples can be applied: whole blood, PRP, platelet concentrates. Allows simultaneous analysis of other parameters such as platelet activation markers.	Measures only intracellular levels of ROS.
2′,7′-dichlorodihydrofluorescein (H2DCFDA) [183,184,185]	Platelets suspensions are incubated with H2DCFDA for 15–20 min at 37 °C.			Measures only intracellular levels of ROS. Require a two-step reaction to detect ROS. Plasma esterases can interfere with the results.
MitoSOX™ Red [94,152,176,186]	Platelets suspensions are incubated with MitoSOX Red for 20–30 min at 37 °C.		Easy, fast and inexpensive way of detecting mitochondrial ROS production in cells.	Detects superoxide but not other ROS or RNS.
Cytochrome C [187,188]	Washed platelets are preincubated with DMSO or inhibitors in the presence of cytochrome C and SOD. Samples are cooled in an ice bath and centrifuged. Reduced cytochrome C is measured in the supernatant.	Spectrophotometry: Absorbance at 550 nm	The method can be performed with equipment available in most laboratories.	Ferricytochrome C can be directly reduced by electrons donated from enzymes and other molecules, so this change in absorbance is not specific for O_2_^·−^. Must be performed in the presence/absence of SOD and only the SOD-inhibitable signal is used to calculate the amount of O_2_^·−^ formed. Only detects extracellular O_2_^·−^. Intracellular sources of O_2_^·−^ are likely underestimated by this method.
Thiobarbituric acid-reactive substances (TBARS) [179,180,181,189]	Washed platelets are preincubated with DMSO or inhibitors in the presence of butylated hydroxytoluene. Then, samples were cooled in an ice bath in the presence of 20% trichloroacetic. Supernatant obtained after centrifugation is mixed with TBA and incubated for 30 min at 70 °C.	Spectrophotometry: Measurement of Malondialdehyde (MDA) produced Absorbance at 532 nm	Marker of lipid peroxidation. Low cost. Easily reproducible	Long sample preparation. Interfering substances. Lack of specificity.
Electron spin resonance (ESR) spectroscopy [134]	For in vivo analysis: fully anesthetized mice after the injection of agonist and radical probe by tail vein injection For in vitro: platelet rich plasma (PRP) Mixed with reaction mixture EMPO and incubated for 2 min before collagen addition.	ESR Spectrometry: In vivo (mid-thorax) and in vitro (PRP) ESR spectra are recorded.	Facilitates the direct identification of transient radicals by stabilizing them using spin-trapping agents like EMPO and nitroxyl radical probes such as CAT1. Robust and non-invasive means to investigate ROS dynamics, kinetics and mechanistic roles during platelet activation	Requires a spectrometer and is essential to have extensive training to operate the equipment
Total antioxidant capacity (TAC) [177,178,179]	Platelet lysate is prepared by homogenizing or sonicating cells in ice-cold 1× PBS and centrifugation for 10 min at 14,000 rpm to pellet any debris. The supernatant is used for the assay.	Measures the inhibition of the absorbance by antioxidants of the radical cation of ATBS•+, which has a characteristic absorption spectrum with maxima at 415, 660, 734 and 820 nm.	Inexpensive and simple method. Detection of both oxidative and reductive stress.	It does not provide information on the nature of the antioxidants present in the sample.

Abbreviations: ATBS: 2,2-azinobis (3-ethylbenzothiazoline 6-sulfonate). CAT1: 4-trimethylammonium-2,2,6,6-tetramethylpiperidine-1-oxyl. EMPO: 5-ethoxycarbonyl-5-methyl-1-pyrroline-N-oxide.

**Table 2:** Agents with redox regulatory activity in platelets used in therapy. Upwards arrows denote increased effect. Downwards arrows denote decreased effect.

Antioxidant	Mechanism	Benefits			References
		Storage	Animal Models	Translational Applications	
**N-acetylcysteine (NAC)**	ROS scavenger. ↑ GSH levels, GPx-1 and SOD1 levels.	Prevents clearance and preserve function of cold-stored platelets.	Attenuates systemic platelet activation and cerebral vessel thrombosis in diabetes ↓ ROS, ↓ mitochondrial ROS, ↓ platelet production and activation in essential thrombocythemia (ET) mouse model.	In vitro treatment of ET patient platelets: ↓ ROS generation, ↓ mitochondrial ROS, ↓ platelet activation and restore platelet function Potential therapeutic strategy for reducing platelet count and restore platelet function in ET.	[19,114,192,195]
**Resveratrol**	Scavenger for intracellular ROS. Inhibits the phosphorylation of the MAPK and activating the cyclic-GMP/vasodilator-stimulated phosphoprotein pathway Inhibition of COX-1.	Inhibits platelet apoptosis and activation during storage.	Suppresses tumor-induced platelet activation in mice with non-small cell lung cancer.	↓ Thrombosis in patients with colon cancer Potential of reducing thrombosis in cancer patients ↓ in vitro thrombus formation and TXA2 formation in human platelets of type 2 diabetic patients Potential to prevent vascular complications diabetic patients	[9,190,198]
**Mito-TEMPO**	Mitochondria-targeted superoxide dismutase antioxidant mimetic.	↓ ROS production ↓ platelet activation Delayed senescence during platelet storage	-	In vitro study (human platelets): ↓ doxorubicin-induced intracellular and mitochondrial ROS generation and prevent platelet apoptosis and GPIbα shedding. Potential clinical application in platelet-associated disorders involving mitochondrial oxidative damage.	[97,187]
***Carica papaya* leaf extract**	Activation of antioxidant enzymes SOD and catalase Membrane stabilizing properties.	↑ the TAC and upregulates the antioxidant enzymes SOD and catalase, while maintaining platelet function in response to agonists.	-	-	[191]
**L-Carnitine**	↓ Lipid peroxidation in platelets ↓ Mitochondrial ROS ↓ Cytochrome C release to cytosol and consequent apoptosis.	Maintain metabolism and antioxidant capacity of PCs and ↓ mitochondrial damage ↓ platelet oxidative stress and platelet apoptosis during storage	-	In patients undergoing major abdominal surgery, ↓ platelet ROS production, ↓ systemic NOX2 activity and ↓ platelet activation Potential therapeutic application in modulating oxidative stress and platelet activation during major abdominal surgery-dependent oxidative damage.	[92,179,206,207]
**NOX-specific inhibitors**	VAS2870: specific inhibition of NOX 1/2 APX-115: Pan-NOX inhibitor	**VAS2870:** preserve mitochondrial function, ↓ apoptosis and ↑ viability of stored platelets. Attenuated PS exposure in stored platelets (>5 days)	**APX-115:** inhibits platelet adhesion and thrombus formation under flow conditions in vitro and arterial thrombosis in vivo (FeCl3-induced carotid artery occlusion model)	Therapeutic potential of APX-115 in treating thrombotic and cardiovascular disorders by targeting NOX-mediated ROS production to mitigate platelet hyperreactivity and thrombus formation.	[21,98]
**Luteolin**	Inhibits the activation of MAPK signaling pathway ↑ SOD and GPx activity ↓ NOXs activity	-	Inhibits thrombus formation in vivo Arterial thrombosis was examined in a mouse model of mesenteric thrombosis induced by ferric chloride	Inhibits GPVI-mediated platelet superoxide production (in vitro), promotes SOD and GPx activity and impairs NOXs activity Potential as antiplatelet agent targeting GPVI	[199]
**Taxifolin**	Regulates the platelet related MAPK and PI3K/Akt pathways	-	Prevents thrombus formation in mice: collagen + adrenaline induced pulmonary embolism and FeCl3 induced carotid artery thrombosis models	In vitro human platelets: inhibited platelet integrin αIIbβ3 ‘’outside-in ‘’ signaling, essential for stable clot formation. Potential as an antiplatelet and antithrombotic agent	[186]
**Cinnamtannin B-1**	ROS scavenger Inhibits oxidants production and Ca^2+^ mobilization evoked by thapsigargin + ionomycin or thrombin	-	-	Antioxidant action in human platelets and reverses the enhanced Ca^2+^ entry and hyperaggregability observed in platelets from type 2 diabetic donors compared with controls. Potential to reduce the risk of thrombotic and ischemic events in diabetic patients.	[200]
**Dimethylthiourea (DMTU)**	Inhibition of collagen-mediated ROS production	-	Attenuated collagen-induced oxidative stress and improved animal survival in collagen-induced thrombosis	Promising therapeutic candidate for preventing thrombotic disorders and managing cardiovascular risk	[134]

## Data Availability

No new data were created or analyzed in this study. Data sharing is not applicable to this article.
